# Psychological distress, health protection, and sexual practices among young men who have sex with men: Using social action theory to guide HIV prevention efforts

**DOI:** 10.1371/journal.pone.0184482

**Published:** 2017-09-08

**Authors:** Ian W. Holloway, Dorian E. Traube, Sheree M. Schrager, Diane Tan, Shannon Dunlap, Michele D. Kipke

**Affiliations:** 1 Department of Social Welfare, Luskin School of Public Affairs, University of California, Los Angeles, Los Angeles, CA, United States of America; 2 Suzanne Dworak-Peck School of Social Work, University of Southern California, Los Angeles, CA, United States of America; 3 Children’s Hospital Los Angeles, Los Angeles, CA, United States of America; 4 Department of Health Policy and Management, UCLA Fielding School of Public Health, Los Angeles, CA, United States of America; University of Toronto, CANADA

## Abstract

The present study addresses gaps in the literature related to theory development for young men who have sex with men (YMSM) sexual practices through the application and modification of Social Action Theory. Data come from the Healthy Young Men study (N = 526), which longitudinally tracked a diverse cohort of YMSM ages 18–24 to characterize risk and protective factors associated with drug use and sexual practices. Structural equation modeling examined the applicability of, and any necessary modifications to a YMSM-focused version of Social Action Theory. The final model displayed excellent fit (CFI = 0.955, TLI = 0.947, RMSEA = 0.037) and suggested concordance between social support and personal capacity for sexual health promotion. For YMSM, practicing health promotion and avoiding practices that may put them at risk for HIV was associated with both social isolation and psychological distress (β = -0.372, t = -4.601, *p*<0.001); psychological distress is an internalized response to environmental and cognitive factors and sexual practices are an externalized response. Results point to the utility of Social Action Theory as a useful model for understanding sexual practices among YMSM, the application of which shows health protective sexual practices are a function of sociocognitive factors that are influenced by environmental contexts. Social Action Theory can help prevention scientists better address the needs of this vulnerable population.

## Introduction

HIV incidence and prevalence among men who have sex with men (MSM) is alarmingly high and increasing prevention efforts for this population are a national priority [1]. MSM have been disproportionately impacted by HIV/AIDS, with transmission in this community accounting for 67% of all new infections, even though MSM represent less than 2% of the general population [2]. Young men who have sex with men (YMSM) are at particular risk for HIV infection [3, 4], with prevalence rates ranging from 2% to 14% across major U.S. metropolitan areas [5–8]. Epidemiological and behavioral research suggests a significant and growing number of YMSM engage in sexual practices that may put them at risk for acquiring HIV despite substantial investments in prevention interventions targeted to this population [6, 7].

There is a dearth of targeted evidence-based interventions for YMSM to help combat these threats to their health and well-being [3, 9–12]. Systematic development of theory has been identified as a crucial step for efficacious behavioral prevention interventions [13], as the design of new prevention efforts that are responsive to the developmental, social, and interpersonal contexts for YMSM requires a theoretical understanding of how these contexts shape their sexual practices. The purpose of the present study is to address gaps in the literature related to theory testing and the documentation of social, behavioral, and demographic risk factors associated with sexual practices among YMSM. Through application and modification of Social Action Theory [14], we will clarify the interplay between environmental and sociocognitive processes affecting sexual practices of YMSM to guide intervention development for this high-priority population.

### Contexts that influence, constrain, facilitate or shape sexual practices among YMSM

Developmental factors are likely to play an important role in the sexual practices and trajectories of YMSM. Late adolescence and early adulthood is a period during in which young people experiment with behaviors that often present increased risk for negative health outcomes (e.g., drug use). This period of ‘emerging adulthood,’ between the ages of 18 and 26 years, is also a time when young people explore new roles and relationships, establish more intimate attachments, initiate sexual relationships, and define their sexual identity, both privately and publicly [15, 16].

Strong evidence suggests that young people experimenting with same-sex relationships are not afforded the same social support as their heterosexual peers [17]. YMSM may experience disapproval, discrimination, and homophobia from their families, peers, racial/ethnic communities, and faith communities; and for YMSM of color, from the predominately white gay community [18]. While connectedness with family has repeatedly been found to be highly protective against drug use and sexual practices that put young people at risk for HIV [19–22], many YMSM find themselves feeling disconnected and isolated from their families due to implicit or explicit disapproval of their sexual identity. At the same time, gay communities may become important sources of social support for YMSM; however, integration into gay male community contexts may also convey risk due to syndemic factors that put young people at risk for HIV [23].

Several studies of adult and young MSM have found that psychological distress is significantly associated with more risky sexual practices [24–26]. Given the high rates of overlapping psychiatric diagnoses among young gay, lesbian, and bisexual populations [27], psychological factors may play a key role in understanding associations with increased engagement in sexual practices that put YMSM at risk for HIV [26]. Data suggest that feelings of self-esteem, self-efficacy, and social support can result in improved mental health for young gay and bisexual men [17] as well as heterosexual emerging adults [28]. Conversely, emerging adults who feel isolated and have a low sense of self-worth and reduced ability to engage in consequential thinking may display poor mental health and subsequent increased risk behavior [29].

#### Theoretical gaps in addressing YMSM sexual practices

Scholars largely agree that HIV interventions should be theoretically driven[30]and several meta-analyses of HIV prevention interventions for adolescents demonstrate a positive association between theory-based interventions and efficacy [31–33]. In a systematic review of HIV interventions for black MSM, nine of the twelve interventions reviewed were identified as being theory-driven [34]. While the importance of theoretically-driven interventions is clear, the theories often used to guide HIV interventions rely heavily on individualistic models of health behavior [30, 34, 35]. For example, in a meta-analysis of HIV intervention studies for adolescents published from 1997 to 2011 [30], the authors note the popularity of the Health Belief Model [36], the Theory of Reasoned Action [37, 38], and Social Cognitive Theory [39]. Designing interventions that are responsive to the developmental, social, and interpersonal contexts for YMSM requires a theoretical understanding of how these contexts influence YMSM’s sexual practices. Although this research has contributed to the understanding of sexual practices among vulnerable populations, these models often focused on a relatively limited array of explanatory constructs and produced contradictory theoretical perspectives [40].

For example, the Health Belief Model and the Theory of Reasoned Action are often criticized for their focus on individual-level processes, without attention to the social environment in which individuals are embedded [41]. These models have the potential to stigmatize individuals through their emphasis on the role of personal responsibility in producing negative health outcomes. Social Cognitive Theory improves upon individualistic health behavior models by explicitly naming the environment as one of three key factors in its model of triadic reciprocal causation, along with personal factors (e.g, cognitive, affective) and behavior. Indeed, Bandura’s work, along with others, arguably gave rise to the concept of social determinants of health, which focuses on the upstream factors (e.g., poverty, housing) that produce negative health outcomes [42]. More holistic models, such as Minority Stress Theory, focus on the unique experiences of sexual and gender minority people, and make major contributions to understanding the multi-level factors that produce health disparities in lesbian, gay, bisexual and transgender people. Minority Stress Theory includes social determinants of health in the form of *distal* factors (e.g., discrimination, victimization) as well as individual-level *proximal* factors and social contextual factors (e.g., community connectedness, affiliation) [43, 44]. However, conceptualizing stress as the major driver of negative health outcomes for LGBT people may de-emphasize the importance of other cognitive and psychological processes that have been shown important in previous research, such as self-efficacy [45–48].

**Fig 1:**
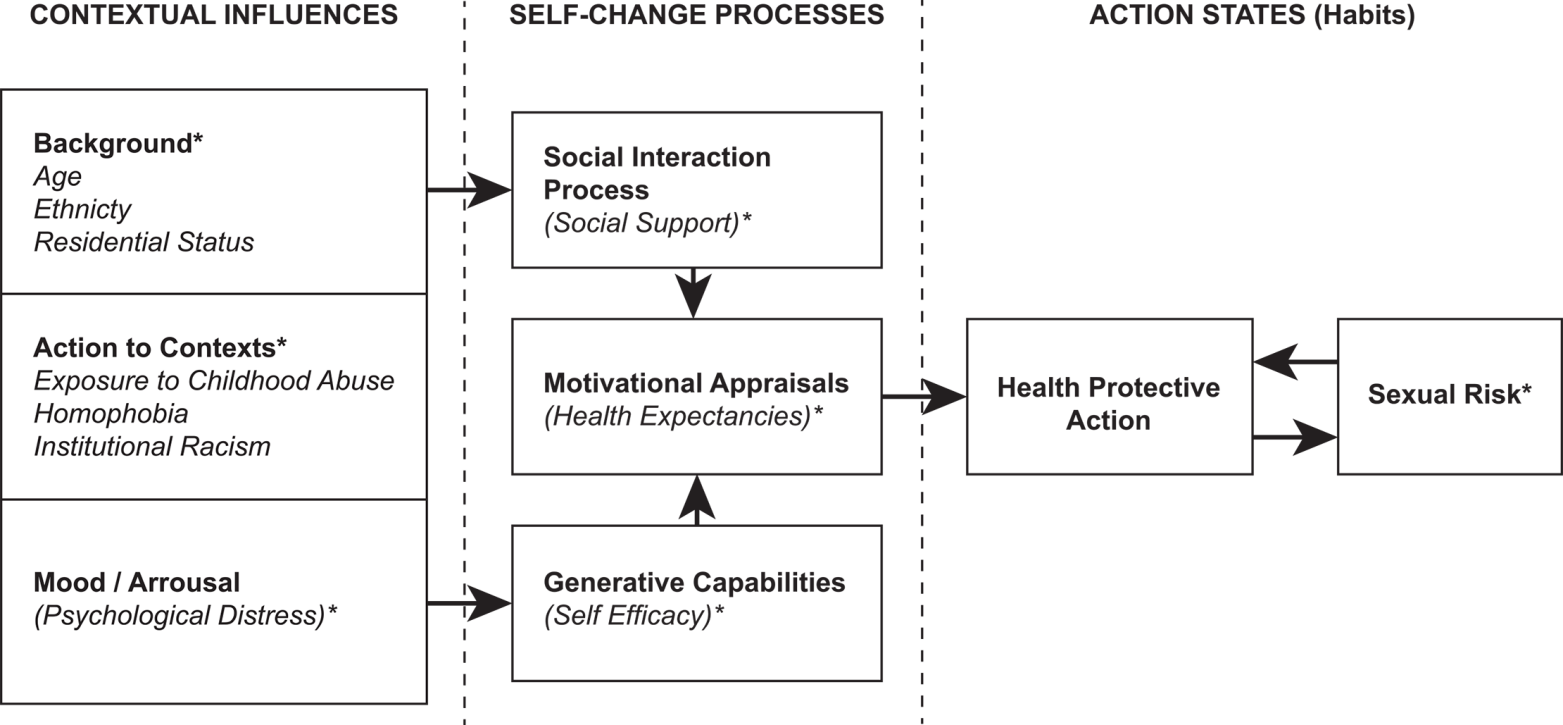
Application of Social Action Theory to sex risk for YMSM. (*) Latent variable title used in current study to aid readability and conceptualization. Adapted from Ewart, 1991; Gore-Felton et al., 2005.

Given the multitude of competing theoretical standpoints, prevention science may benefit from a comprehensive and well-integrated theory that captures and conceptualizes the range of factors associated with sexual risk-taking among YMSM. Social Action Theory [14] holds great promise to this end [49–51]. Developed as a health promotion theory for behavioral medicine, Social Action Theory extends existing sexual health-related sociocognitive models [52–55] by specifically targeting contextual influences on sexual practices. As depicted in Fig 1, Social Action Theory proposes that health protective behaviors are a result of interaction among three domains: 1) contextual influences, such as background, demographics, life stressors, and mental health factors including positive and negative affect; 2) self-change processes, such as social support, health expectancies, and self-efficacy; and 3) action states, including health behavior outcomes such sexual practices. As Ewart originally conceptualized the model, a person’s contextual influences (action contexts) impact their self-change processes (i.e., social support, health expectancies, self-efficacy), which in turn impact their actual health behavior or action [14].

The relevance of Social Action Theory to HIV prevention among YMSM is unique and important because it focuses on the complex interplay between environment, social support, cognitions, and mental health. Additionally, this theory extends beyond previous health behavior theories in describing a vast array of constructs (e.g., motivational influences, generative capabilities), which is especially useful given the multiple determinants of sexual practices for YMSM. Despite this, few HIV prevention interventions targeting YMSM and adolescents in general have made use of this valuable theory [30, 34, 35]. Of the few studies that have used Social Action Theory, few have involved HIV-negative YMSM specifically. Instead, many studies using Social Action Theory have focused on youth and adults living with HIV/AIDS with the aim of identifying factors associated with HIV transmission risk behavior, disclosure, treatment adherence [56–63]. Two studies using Social Action Theory involving high-risk, HIV-negative individuals focused on sexual risk behaviors among adults [64, 65].

While a comprehensive model focusing on environmental, social, and cognitive factors has the potential to make a significant contribution to our understanding of the pathways to HIV transmission among YMSM, more information is needed about the associations and pathways between environmental, psychological, and social factors that contribute to the difficulties faced by sexual minority youth (for an in-depth discussion of the merit of Social Action Theory versus other behavioral health theories, see Traube, Holloway, & Smith, 2011) [66]. However, Social Action Theory requires modification to resolve some inconsistencies specific to emerging adult development. Specifically, the role of mental health in Social Action Theory contradicts relationships reflected in the literature where mental health and subsequent sexual practices that may put individuals at risk for acquiring STIs, including HIV, is depicted as an outcome of stressful life events and poor coping mechanisms, rather than a precursor to such events, for emerging adults. In the original Social Action Theory model, psychological and mental health variables were termed “mood/arousal” and positioned as contextual influences on self-change processes. Mood and arousal were believed to influence the retention of various types of health information as well as behavioral control. Emotional arousal was postulated to effect attention deployment such that under high arousal, individuals would be less able to detect stimuli, attend to their own behavior, or appraise the long-term consequences of personal decisions [14, 67]. Emotional distress was also thought to impair interpersonal problem-solving capabilities [68, 69], and behavioral control [70].

Recent theoretical models highlight the importance of underlying cognitive processes that might be responsible for elevated and persistent depression and poor mental health outcomes [71]. Thus, the present model positions psychological distress as a higher-level outcome of the cognitive processes specified by Social Action Theory rather than an online indicator of emotional status likely to interfere with decision-making on a moment-to-moment basis [72]. This paper explicitly considers health protective behavior, mental health, and sexual practices as outcomes of the contextual factors and self-change processes posited by Social Action Theory. The present analysis intends to test a model grounded in a modified version of Social Action Theory, previously established for YMSM substance use [73], to examine YMSM sexual risk outcomes.

## Methods

The present study is a secondary analysis of baseline data from the Healthy Young Men (HYM) study, a longitudinal study of substance use and sexual practice among YMSM in Los Angeles, California (for a full description, see Ford et al., 2009; Kipke et al., 2007) [74, 75].

### Participants

A total of 526 subjects were recruited into the HYM study. Young men were eligible to participate if they were: male; 18 to 24 years old; self-identified as gay, bisexual, or uncertain about their sexual orientation and/or reported having had sex with another man; a resident of Los Angeles County and anticipated living in Los Angeles County for at least six months; and self-identified as Caucasian, African American, or Latino of Mexican descent.

### Procedures

YMSM were recruited at public venues using the stratified probability sampling design developed by the Young Men’s Study [76] and later modified by the Community Intervention Trials for Youth [77]. Study participants were recruited from 36 different public venues previously identified as settings frequented by YMSM. The survey was administered at a location convenient to the participant, either the project office or a public venue that provided Internet connectivity. The survey was administered in both English and Spanish using computer-assisted interview technologies that incorporated sound files, allowing participants to read questions on the computer while listening to the questions through headphones. Participants entered their responses directly into the computer. The survey took approximately 90 minutes to complete, and participants received $35 as compensation for their time and effort. This research was approved by the Committee on Clinical Investigations at Children’s Hospital Los Angeles; secondary data analysis was approved by the Institutional Review Board of the University of California, Los Angeles.

### Measures

#### Sexual practices

The survey asked respondents about their sexual activity during the past three months, including number of sexual partners, if they had engaged in anal insertive and/or receptive sex, and condom use. Responses were combined to form a four-level index of sexual risk: 1 = no partners, 2 = consistent condom use for anal intercourse, 3 = insertive or receptive condomless anal intercourse with a single seroconcordant partner (here referring to both partners being HIV-negative), and 4 = condomless anal intercourse with a single serodiscordant partner or multiple partners of any HIV status.

#### Psychological distress latent factor

Respondents completed the Centers for Epidemiologic Studies Depression Scale (CES-D) as an indicator of their psychological distress [78]. Participants reported how often they had experienced up to 20 depressive symptoms within the past week (α = .90). A total score was calculated by summing the items. Stressful life events were measured by asking participants if they had experienced stressful events during the previous three months using an instrument developed with an adult MSM population [79] and adapted for YMSM by adding items generated from qualitative data during the formative phase of the HYM study. Thirty binary items assessed the presence of a variety of stressors (e.g., family-related, partner-related, death-related, school/work-related, health-related, finance-related, physical/emotional threat-related, and sexuality-related), and responses were summed to indicate the participant’s aggregate level of life stress. Two additional binary items (1 = yes, 0 = no) assessed whether, in the past 12 months, participants had seriously considered attempting suicide and/or felt so much hopelessness over a two-week period that they ceased their usual activities.

#### Health protection latent factor

Health-related measures included number of days in the past week that participants had engaged in exercise or physical activity [80]. Additionally, a cigarette use index was created from a series of questions ascertaining participants’ history of smoking cigarettes (1 = lifetime non-users, 2 = prior users who had smoked previously but not in the past 30 days, 3 = light users who smoked 15 days or fewer in the past month and 1/2 a pack of cigarettes per day or less, and 4 = frequent or heavy users who smoked more than 15 days in the past month or more than 1/2 a pack of cigarettes per day). For inclusion in the health protection latent factor, the cigarette use index was recoded so that higher scores represented healthier behavior (i.e., less cigarette use).

#### Action contexts latent factor

Two binary measures (1 = yes, 0 = no) of exposure to abuse growing up assessed whether the participant had either witnessed or experienced physical abuse. Experiences of homophobia were measured with a single item asking respondents how often they had to pretend to be heterosexual in order to be accepted. Three items measured experiences of institutional forms of racism (e.g., “How often have you been turned down for a job because of your race or ethnicity?”; α = 0.70), and six items measured experiences of social/sexual racism (e.g., experiences of racism in gay social settings and/or sexual relationships; α = 0.81). All participants completed the racism measures, regardless of self-reported racial or ethnic group. Items measuring experiences of homophobia and racism used a four-point response format (1 = never, 2 = once or twice, 3 = a few times, 4 = many times).

#### Social support latent factor

Four items assessed family support (e.g., “I get the emotional help and support I need from my family”; α = 0.87), and four items assessed perceived friend support (e.g., “I can count on my friends when things go wrong”; α = 0.85). Both support scales were assessed with Likert-type items ranging from 1 = strongly disagree to 4 = strongly agree. Four additional binary items (1 = true, 0 = false) assessed the family’s closeness (e.g., “My family provides me with a lot of support”; α = 0.69).

#### Self-efficacy latent factor

A nine-item, four-point scale assessed participants’ self-efficacy for condom use, such as wearing a condom comfortably or discussing condom use with a partner (1 = couldn’t do it, 2 = unsure, 3 = sure, 4 = very sure; α = 0.79). A separate nine-item, three-point scale assessed endorsement of protective strategies for condom use in specific situations, such as with an HIV-positive partner, when drunk or high, or when having just met a sexual partner the same night (1 = not use a condom, 2 = maybe use a condom, 3 = definitely use a condom; α = 0.88).

#### Health expectancies latent factor

Five items assessing mean risk levels measured the number of the participants’ close friends (up to five) who had tried drugs, used drugs at least once a week, smoked marijuana regularly, used drugs prior to or during sex, and/or drank alcohol prior to or during sex. This measure was reverse-scored such that higher scores represented fewer friends engaging in risk. Three items assessed health as a value to the participant (e.g., “I am willing to make sacrifices to be healthy”; α = 0.67) on a four-point Likert-type scale with response range 1 = not true to 4 = completely true. Finally, health risk health expectancies were measured with five items assessing the participant’s perceived risk for HIV infection, herpes infection, hepatitis infection, drug addiction, and alcoholism compared to other YMSM of the same age (α = 0.82). This measure was also reverse-scored such that higher scores represented more positive health outcome expectancies.

#### Covariates: Sociodemographic measures

Dummy codes represented the respondents’ racial/ethnic category (African American: African American = 1, White = 0, Latino = 0; Latino: Latino = 1, White = 0, African American = 0), residential status (living at home with family = 1, other = 0), and a single continuous variable represented subjects’ age in years.

### Analytic strategies

Structural equation modeling (SEM) with latent variables, a multivariate technique often used to test theoretical models, was the overarching approach to analysis [81, 82]. When the associations among underlying theoretical constructs are represented by latent variables, modeling may decrease measurement error present when using single indicator variables, such as in path analysis. Therefore, SEM may provide more reliable results that reveal relationships among the theoretical constructs, which in turn account for covariation among the individual measured variables. In the present study, the latent variables described in Ewart’s original text on Social Action Theory were created using the pre-existing variables from the HYM study data described above.

The analytic process began with confirmatory factor analysis (CFA) to verify that the indicators loaded as expected onto concise, interpretable latent factors consistent with Social Action Theory constructs (the ‘measurement model’). Subsequent regression paths were systematically added among the established latent variables and the (observed) sexual risk variable to investigate the interrelationships among Social Action Theory constructs and their effects on participants’ sexual practices. Nonsignificant paths were removed until the most parsimonious model was reached (the ‘structural model’). All analyses including imputation of missing data were conducted using full information maximum likelihood estimation in Mplus [83].

## Results

### CFA/Measurement model

Table 1 reports the means, standard deviations, and ranges for the measured variables. All proposed indicator variables loaded significantly (*p* < 0.001) on their hypothesized latent factors. Residual covariances were added between (1) the indicators representing violence and abuse histories in the action contexts latent variable, (2) the institutional racism and social/sexual racism indicators of the action contexts latent variable, and (3) the suicidality and hopelessness indicators of the psychological distress latent variable. The final measurement model displayed excellent fit (CFI = 0.963, TLI = 0.953, RMSEA = 0.038, χ^2^(171) *=* 2933.56, p<0.001). Nearly two-thirds (64%) of participants did not indicate serodiscordant or multiple partners, giving us very good distribution on our primary analytical outcome.

**Table 1:** Descriptive statistics for observed and latent variables in the structural model.

Variables	Mean (SD) *or* N (%)
*Observed Variables*	
Age in years (range: 18–24)	20.14 (1.58)
Residential status–live with family	281 (53%)
Race/ethnicity:	
African American	125 (24%)
Latino of Mexican descent	205 (39%)
White	195 (27%)
Sexual behavior:	
No partners	86 (18%)
Protected AI	216 (46%)
Single-partner seroconcordant UAI	45 (10%)
Serodiscordant or multiple partners UAI	125 (26%)
*Latent variables*	
Action contexts	
Social/sexual racism (range: 1–4)	1.64 (0.58)
Institutional racism (range: 1–4)	1.45 (0.61)
History of witnessing physical abuse (binary)	104 (20%)
History of experiencing physical abuse (binary)	133 (25%)
Experiences of homophobia (range: 1–4)	2.50 (1.17)
Health expectancies	
Low friends’ sexual/drug use practices (range: 1–4)*	3.14 (0.65)
Health values (range: 1–4)	3.44 (0.56)
Low health risk expectancies (range: 1–5)*	3.97 (0.81)
Self-efficacy	
Self-efficacy for HIV prevention (range: 10–32)	28.52 (3.79)
Protective strategies (range: 1–3)	2.57 (0.44)
Social support	
Family support (range: 1–4)	3.00 (0.71)
Friend support (range: 1–4)	3.51 (0.51)
Family closeness (range: 0–4)	3.21 (1.14)
Health protective behaviors	
Exercise (range: 1–8)	3.81 (2.04)
Less cigarette smoking (range: 1–4)*	2.61 (1.16)
Psychological distress	
Depressive symptoms score (range: 0–54)	15.04 (10.55)
Stressful life events (range: 0–21)	6.53 (3.93)
Suicidality (binary)	53 (10%)
Hopelessness (binary)	160 (31%)

*Variables are reverse coded so that higher values indicate health protective constructs.

### Structural model

**Fig 2:**
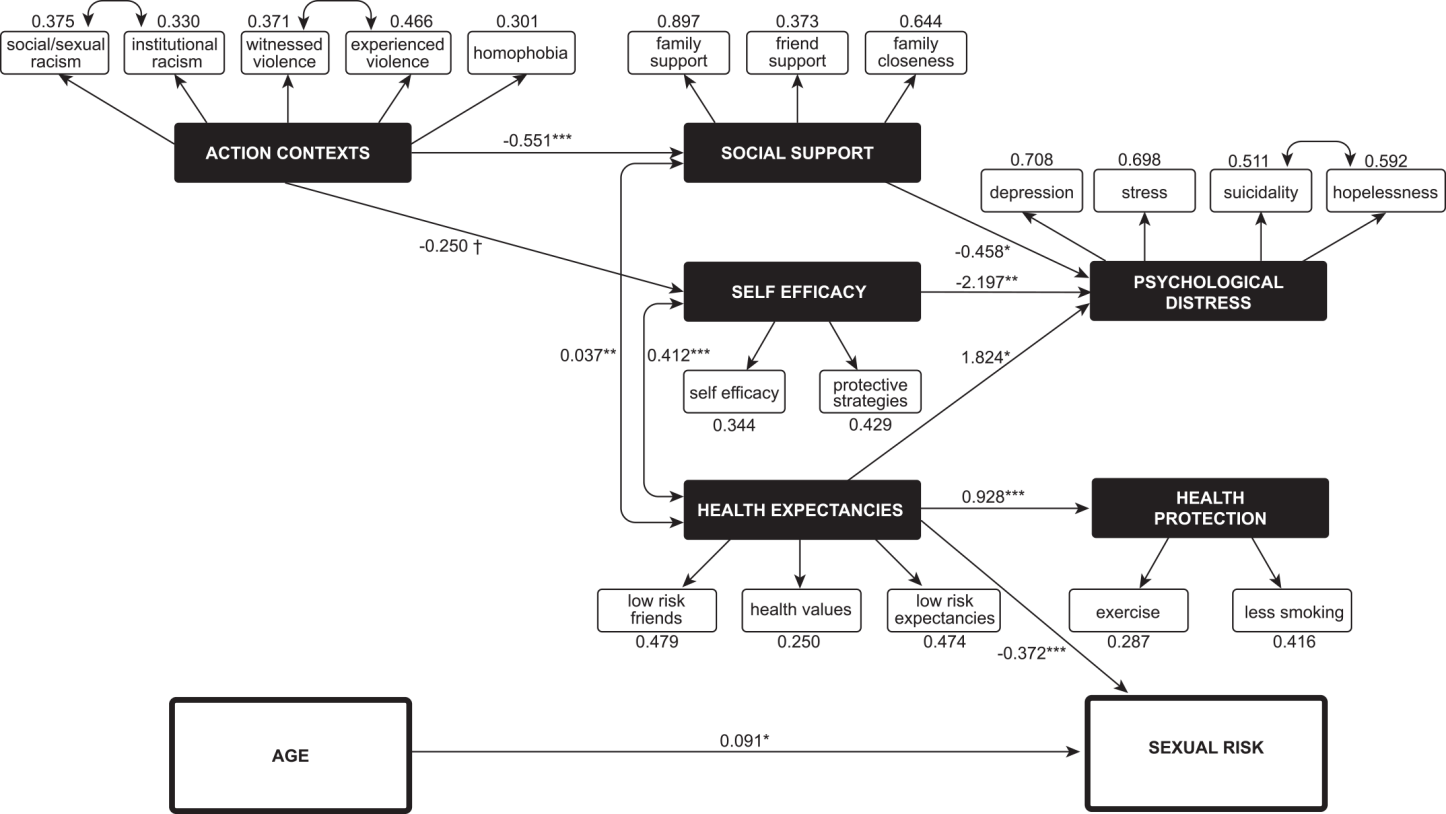
Social Action Theory variables predicting sexual risk among YMSM. (ϯ) p < 0.10. (*) p < 0.05. (**) p < 0.01. (***) p < 0.01.

The model predicting sexual risk, presented in Fig 2, also displayed excellent fit and good explanatory power for sexual risk (CFI = 0.955, TLI = 0.947, RMSEA = 0.037; χ^2^(178) = 304.85, p<0.001, R^2^ = 0.15). Two statistically significant associations between latent variables emerged: health expectancies were positively associated with self-efficacy (r = 0.412, *p*<0.001) and social support (r = 0.037, *p*<0.01). Significant latent variable covariances between these intermediary constructs indicated shared variance between health expectancies, self-efficacy, and social support.

Action contexts were significantly negatively associated with social support (β = -0.551, t = -3.839, *p*<0.001) and marginally associated with self-efficacy (β = -0.250, t = -1.819, *p*<0.10), suggesting that participants with greater experiences of violence and discrimination had both lowered perceptions of social support and less comfort with condom use as an HIV prevention strategy. Social support (β = -0.458, t = -1.999, *p*<0.05) and self-efficacy (β = -2.197, t = -2.580, *p*<0.01) were in turn significantly associated with reduced psychological distress, suggesting concordance between social support, personal capacity for HIV prevention, and mental health. Health expectancies, on the other hand, were positively related to psychological distress (β = 1.824, t = 2.310, *p*<0.05), suggesting that motivations to avoid friends who engage in sexual and drug use practices associated with HIV transmission may have consequences to psychological wellbeing. However, health expectancies were positively related to health protective behaviors (β = 0.928, t = 4.170, *p*<0.001) and negatively related to risk practices associated with HIV transmission (β = -0.372, t = -4.601, *p*<0.001). These findings suggest a difference in risk factors for mental versus physical health, as positive health motivations were predictive of healthier behaviors, including lowered likelihood of engagement in risky sexual practices, despite being associated with increased psychological distress. Finally, age was significantly associated with sexual practices associated with HIV (β = 0.091, t = 2.009, *p*<0.05), such that older participants were more likely to engage in sexual practices that may put them at risk for HIV acquisition. Race/ethnicity and residential status were also tested for association with sexual practices; however, neither were significant predictors of sexual practices and were excluded from the final model. In accordance with the Social Action Theory framework, the relationship between psychological distress and sexual risk was also examined; however, no association was found between these outcomes in our sample.

## Discussion

The purpose of the present study was to test a model grounded in a modified version of Social Action Theory [14] to address the individual, contextual, and social correlates of sexual practices of YMSM. Below we examine each segment of the model to interpret these complex findings for this under-studied population at high risk for HIV infection. It is important to note that nearly two-thirds of participants did not report engaging in the highest-risk sexual practices (condomless anal intercourse with a single serodiscordant partner or multiple partners of any HIV status). Our primary analytical outcome is nicely distributed for the types of analyses presented in this report, and reflects the full range of HIV risk experienced by YMSM in our study–including the lower-risk (but not perfectly protective) category of protected anal intercourse, a category representing 46% of our participants. We are thus able to model a more sensitive measure of sexual risk than found in prior work that relied on dichotomous indicators of risk, for example, a binary condom use measure without regard for number or type of partner(s). We believe the distribution on our primary analytical outcome strengthens our results by allowing for the interpretation of regression coefficients and associated predicted values along a continuum of risk.

### Contextual influences

According to Ewart’s conceptualization of Social Action Theory [14], individual behavior is based on the normative expectations of social settings comprised of background variables, action contexts, and psychological distress.

#### Background variables

In this study, age was the only significant background, or demographic, variable related to sexual practices. Older participants were more likely to engage in riskier sexual practices, which is consistent with findings reported about heterosexual adolescent and young adult sexual practices in the United States [5, 84]. Contrary to other epidemiological surveys [85], race/ethnicity and residential status were not significantly associated with sexual practices associated with HIV and were excluded from the final model.

#### Action contexts

Action contexts refer to the societal and institutional milieu which support or hinder the maintenance of health routines or habits. Action contexts were significantly negatively associated with the social support latent variable and marginally associated with self-efficacy, a cognitive mediating variable representing an individual’s perceived ability to protect himself against HIV. This suggests that participants with greater experiences of violence and discrimination had both lowered perceptions of social support and less confidence in their abilities to use condoms as an HIV prevention strategy [86]. The variable representing homophobia as part of action contexts partially accounts for sexual stigma that is often faced by YMSM [87]. Minority Stress Theory conceptualizes multiple forms of sexual stigma as a key driver of health disparities among LGBT people [43, 44] and further work warrants additional integration of sexual stigma into action contexts, which may bolster the importance of action contexts on individual-level sociocognitive processes.

### Self-change processes

In Social Action Theory, the outcome variables of interest–action states comprised of health risk and protective behaviors–arise from strategies individuals use when trying to regulate their behavior. The creation of these behaviors is prompted by the ability to make assessments (i.e., health expectancies) and translate them into strategies that are a function of health-relevant procedural and factual knowledge (i.e., self-efficacy) as well as the social support provided by those who influence the individual (i.e., social support) [14]. The constructs of self-efficacy and health expectancies are both key elements of Social Cognitive Theory and have been associated with sexual practices among YMSM [52]. In Social Action these individual-level cognitive processes influence behavioral health outcomes and are influenced by action contexts.

#### Social support and self-efficacy

In our test of Social Action Theory, increased social support and self-efficacy was associated with lower levels of psychological distress, suggesting a relationship between social support, personal strategies for HIV prevention, and mental health. Given that YMSM who have supportive family and friends report less psychological distress, family- and peer-based interventions for this population may be useful. Additionally, interventions that increase self-efficacy and protective strategies may have the added benefit of reducing psychological distress as well as increasing safer sexual practices.

#### Health expectancies

Likewise, health expectancies were positively associated with health protective behaviors and negatively associated with risky sexual practices. However, individuals with greater health expectancies experienced more psychological distress. These findings suggest a difference in risk factors for mental versus physical health, as positive health motivations were related to healthier behaviors, including lower sexual practices that put YMSM at risk for HIV, despite being associated with an increase in psychological distress. One explanation for these seemingly contradictory findings may relate to the fact that while MSM communities have potential for supportive interaction, they also carry significant risks based on high base-rates of sexual practices and social norms which may put YMSM at risk for HIV [23]. When YMSM attempt to engage in health promotion they may need to avoid social networks where sexual practices that carry HIV risk are normative. Finally, health expectancies covaried with self-efficacy and social support, suggesting that the relationship between health expectancies and sexual practices is informed by the other self-change processes. These findings further support and validate the multidimensional targeting of behavior, which has been shown to successfully predict behavioral health outcomes [61, 88].

#### Action states

Considering the associations between intermediary self-change processes and the outcome variables within action states, the results of this study provide general support for Ewart’s model for determining pathways to health protective behaviors and sexual practices that may put YMSM at risk for HIV. This model is more salient to the needs of YMSM when psychological distress is included as an action state. Specifically, psychological distress would be considered an internalizing response to environmental and cognitive factors while sexual practices would be an externalizing response. The application of Social Action Theory to determining sexual practices of YMSM shows that health protective behavior is a function of sociocognitive factors that are influenced by environmental contexts. For YMSM, the choice of practicing health promotion and avoiding sexual practices that put them at risk for HIV may be related to both social isolation and psychological distress. Interestingly, psychological distress was not statistically significantly associated with sexual risk behavior. This null finding is consistent with other studies involving people living with HIV, including a recent multisite study that used Social Action Theory to understand sexual transmission risk [58], and studies involving at-risk adolescents [89–93]. One explanation for this may be our operationalization of psychological distress, which included relatively severe indicators of poor mental health (e.g., depression, suicidality, hopelessness). Those experiencing these issues may be more socially isolated and have fewer opportunities to engage in sexual practices. This relationship warrants additional attention in future research and others may consider a more expansive operationalization of psychological distress or may seek to explore specific mental health issues in relation to sexual practices that may put YMSM at risk for HIV.

### Strengths and limitations

The results of the present study are based on a large probability sample of YMSM and offer an opportunity, not previously possible, to empirically validate Social Action Theory with a population of sexual minority youth. The importance of this research rests on its potential to further the understanding of these and other factors that influence sexual practices among YMSM in order to enhance behavioral prevention efforts with this population.

Despite these strengths, this study has some limitations. Due to issues of statistical power, this study is cross-sectional. Future studies should explore trajectories of sexual practices for YMSM, particularly given the volatility of the developmental period of emerging adulthood. Inquiry into the role of mental health symptoms as a precursor to, outcome of, or simultaneous force acting on both sides of the sociocognitive factors producing sexual practices has the potential to be particularly impactful. The data for this study come from a larger study designed to characterize the individual-, familial-, social-, and community-level factors associated with drug use and sexual practices of YMSM. Therefore, the test of the application of Social Action Theory is limited by the use of secondary data that was not gathered for the specific purpose of empirically validating Social Action Theory. For example, we were limited to a single item to measure internalized homophobia, one dimension of the complex psychological construct known as sexual stigma, which is not ideal. Future studies seeking to apply Social Action Theory to health practices with vulnerable populations should be designed, with the intention of testing the theory, and target data collection based on the exact specification of the domains in this theory as originally conceptualized by Ewart [14].

Data were collected from participants at gay-identified venues; therefore the data may not generalize to men who do not attend these venues, especially those who do not openly identify as gay or bisexual. The study data were gathered by self-report and may underestimate the true prevalence of sexual practices that put YMSM at risk for HIV, given that those practices may be perceived by many as socially undesirable. However, computer assisted interviewing technology may have helped minimize this bias.

## Conclusion

Results from this study have important implications for the field of sexual health promotion, particularly efforts to create theoretically-driven interventions to prevent sexual practices that put YMSM at risk for HIV. Findings support the utility of Social Action Theory as a theoretical model for understanding sexual practices of YMSM. The model presented here highlights the unique relationships between environmental experiences, individual self-change processes, and mental health and sexual practices. Therefore, behavioral prevention interventions for this population may benefit from employing Social Action Theory and using a multi-targeted strategy for impacting several predictors of sexual practices among YMSM, particularly limited social support and psychological distress. Given the lack of inquiry into targeted theories for YMSM, this cross-sectional study can serve as a foundation for developing even more robust empirically-validated health behavior theories for this population.

In sum, efficacious behavioral prevention intervention development is particularly important for YMSM given that they are at a distinctive developmental time point between adolescence and adulthood marked by increased vulnerability and sexual practices that may put them at risk for HIV. Social Action Theory is especially useful for framing YMSM behavioral prevention interventions because it emphasizes the social context and its effect on the individual-level processes that lead to sexual practices that may put YMSM at risk for HIV.

## References

[1] White House Office of National AIDS Policy. National HIV/AIDS Strategy for the United States: Updated to 2020. In: House W, editor. Washington, DC2015.

[2] Centers for Disease Control. HIV among Gay and Bisexual Men 2016 [cited 2016 May 12]. Available from: http://www.cdc.gov/hiv/group/msm/.

[3] Mustanski BS, Newcomb ME, Du Bois SN, Garcia SC, Grov C. HIV in young men who have sex with men: a review of epidemiology, risk and protective factors, and interventions. Journal of sex research. 2011;48(2–3):218–53. Epub 2011/03/17. 10.1080/00224499.2011.558645 21409715 PMC3351087

[4] Rotheram-Borus M, O'Keefe Z, Kracker R, Foo H. Prevention of HIV among adolescents. Prevention Science. 2000;1(1):15–30. 11507791 10.1023/a:1010071932238

[5] Centers for Disease Control. HIV Surveillance Report, 2008. 2010 June 2010. Report No.

[6] MacKellar DA, Valleroy LA, Secura GM, Behel S, Bingham T, Celentano DD, et al Unrecognized HIV Infection, Risk Behaviors, and Perceptions of Risk Among Young Men Who Have Sex With Men: Opportunities for Advancing HIV Prevention in the Third Decade of HIV/AIDS. Journal of Acquired Immune Deficiency Syndromes. 2005;38:603–14. 15793373 10.1097/01.qai.0000141481.48348.7e

[7] Sifakis F, Hylton JB, Flynn C, Solomon L, MacKellar DA, Valleroy LA, et al Prevalence of HIV infection and prior HIV testing among young men who have sex with men. The Baltimore young men's survey. AIDS Behav. 2010;14(4):904–12. 10.1007/s10461-007-9317-5 . 17968648

[8] Centers for Disease Control. HIV Surveillance Report, 2014. 2014;26.

[9] Bauermeister JA, Pingel ES, Jadwin-Cakmak L, Harper GW, Horvath K, Weiss G, et al Acceptability and preliminary efficacy of a tailored online HIV/STI testing intervention for young men who have sex with men: the Get Connected! program. AIDS Behav. 2015;19(10):1860–74. Epub 2015/02/02. 10.1007/s10461-015-1009-y 25638038 PMC4522230

[10] Hosek SG, Siberry G, Bell M, Lally M, Kapogiannis B, Green K, et al The acceptability and feasibility of an HIV preexposure prophylaxis (PrEP) trial with young men who have sex with men. Journal of acquired immune deficiency syndromes (1999). 2013;62(4):447–56. Epub 2013/10/19. 10.1097/QAI.0b013e3182801081 24135734 PMC3656981

[11] Johnson WD, Holtgrave DR, McClellan WM, Flanders WD, Hill AN, Goodman M. HIV intervention research for men who have sex with men: A 7-year update. AIDS Education and Prevention. 2005;17(6):568–89. 10.1521/aeap.2005.17.6.568 16398578

[12] Mustanski BS, Garofalo R, Monahan C, Gratzer B, Andrews R. Feasibility, acceptability, and preliminary efficacy of an online HIV prevention program for diverse young men who have sex with men: the keep it up! intervention. AIDS Behav. 2013;17(9):2999–3012. Epub 2013/05/16. 10.1007/s10461-013-0507-z 23673793 PMC3796205

[13] Rounsaville BJ, Carroll KM, Onken LS. A stage model of behavioral therapies research: Getting started and moving on from Stage I. Clinical Psychology: Science and Practice. 2001;8(2):133–42.

[14] Ewart CK. Social Action Theory for a public health psychology. American Psychologist. 1991;46:931–6. 1958012 10.1037//0003-066x.46.9.931

[15] Arnett JJ. Emerging adulthood: A theory of development from the late teens through the twenties. American Psychologist. 2000;55(5):469–80. 10.1037/0003-066x.55.5.469 10842426

[16] Laska MN, Pasch KE, Lust K, Story M, Ehlinger E. Latent class analysis of lifestyle characteristics and health risk behaviors among college youth. Prev Sci. 2009;10(4):376–86. 10.1007/s11121-009-0140-2 19499339 PMC2927491

[17] Rosario M, Schrimshaw EW, Hunter J. A model of sexual risk behaviors among young gay and bisexual men: longitudinal associations of mental health, substance abuse, sexual abuse, and the coming-out process. AIDS Educ Prev. 2006;18(5):444–60. 10.1521/aeap.2006.18.5.444 17067255 PMC3222951

[18] Huebner DM, Davis MC. Perceived antigay discrimination and physical health outcomes. Health Psychology. 2007;26(5):627–34. 10.1037/0278-6133.26.5.627 17845114

[19] McConnell EA, Birkett MA, Mustanski B. Typologies of Social Support and Associations with Mental Health Outcomes Among LGBT Youth. LGBT health. 2015;2(1):55–61. Epub 2016/01/21. 10.1089/lgbt.2014.0051 . 26790019 PMC4855776

[20] Needham BL, Austin EL. Sexual orientation, parental support, and health during the transition to young adulthood. Journal of youth and adolescence. 2010;39(10):1189–98. Epub 2010/04/13. 10.1007/s10964-010-9533-6 . 20383570

[21] Rosenblum A, Magura S, Fong C, Cleland C, Norwood C, Casella D, et al Substance Use Among Young Adolescents in HIV-Affected Families: Resiliency, Peer Deviance, and Family Functioning. Substance Use & Misuse. 2009;40(5):581–603. 10.1081/ja-200030816 15887592

[22] Ryan C, Russell ST, Huebner D, Diaz R, Sanchez J. Family acceptance in adolescence and the health of LGBT young adults. Journal of child and adolescent psychiatric nursing: official publication of the Association of Child and Adolescent Psychiatric Nurses, Inc. 2010;23(4):205–13. Epub 2010/11/16. 10.1111/j.1744-6171.2010.00246.x . 21073595

[23] Stall R, Friedman M, Catania JA. Interacting epidemics and gay men’s health: A theory of syndemic production among urban gay men In: Wolitski R. J., Stall R., Valdesseri RO, editors. Unequal opportunity: Health disparities affecting gay and bisexual men in the United States. New York City, NY: Oxford University Press; 2008 p. 251–74.

[24] Wong CF, Kipke MD, Weiss G, McDavitt B. The impact of recent stressful experiences on HIV-risk related behaviors. Journal of adolescence. 2010;33(3):463–75. Epub 2009/07/18. 10.1016/j.adolescence.2009.06.004 19608264 PMC2862810

[25] Safren SA, Reisner SL, Herrick A, Mimiaga MJ, Stall R. Mental Health and HIV Risk in Men Who Have Sex with Men. Journal of acquired immune deficiency syndromes (1999). 2010;55(Suppl 2):S74–S7. 10.1097/QAI.0b013e3181fbc939 PMC3074520. 21406991 PMC3074520

[26] Mustanski BS. The influence of state and trait affect on HIV risk behaviors: A daily diary study of MSM. Health Psychology. 2007;26(5):618–26. 10.1037/0278-6133.26.5.618 17845113

[27] Bontempo DE, D’Augelli AR. Effects of at-school victimization and sexual orientation on lesbian, gay, or bisexual youths’ health risk behavior. Journal of Adolescent Health. 2002;30(5):364–74. 10.1016/S1054-139X(01)00415-3. 11996785

[28] Patel V, Flisher AJ, Hetrick S, McGorry P. Mental health of young people: a global public-health challenge. The Lancet. 2007;369(9569):1302–13. 10.1016/s0140-6736(07)60368-7 17434406

[29] Clark LF, Miller KS, Nagy SS, Avery J, Roth DL, Liddon N, et al Adult identity mentoring: Reducing sexual risk for African-American seventh grade students. Journal of Adolescent Health. 2005;37(4):337.e1-.e10. 10.1016/j.jadohealth.2004.09.024. 16182145

[30] Protogerou C, Johnson BT. Factors underlying the success of behavioral HIV-prevention interventions for adolescents: A meta-review. AIDS and Behavior. 2014;18(10):16. 10.1007/s10461-014-0807-y 24903669

[31] Shepherd J, Kavanagh J, Picot J, Cooper K, Harden A, Barnett-Page E, et al The effectiveness and cost-effectiveness of behavioural interventions for the prevention of sexually transmitted infections in young people aged 13–19: a systematic review and economic evaluation. Health technology assessment (Winchester, England). 2010;14(7):1–206, iii-iv. Epub 2010/02/25. 10.3310/hta14070 . 20178696

[32] Levin KD. Preventing sexually transmitted HIV infection in adolescents: Predicting condom use behaviors and reducing risk: Syracuse University; 2002.

[33] Kim N, Stanton B, Li X, Dickersin K, Galbraith J. Effectiveness of the 40 adolescent AIDS-risk reduction interventions: a quantitative review. The Journal of adolescent health: official publication of the Society for Adolescent Medicine. 1997;20(3):204–15. Epub 1997/03/01. 10.1016/s1054-139x(96)00169-3 . 9069021

[34] Maulsby C, Millett G, Lindsey K, Kelley R, Johnson K, Montoya D, et al A systematic review of HIV interventions for black men who have sex with men (MSM). BMC Public Health. 2013;13(1):625 10.1186/1471-2458-13-625 23819660 PMC3710496

[35] Lorimer K, Kidd L, Lawrence M, McPherson K, Cayless S, Cornish F. Systematic review of reviews of behavioural HIV prevention interventions among men who have sex with men. AIDS care. 2013;25(2):133–50. Epub 2012/07/11. 10.1080/09540121.2012.699672 . 22774763

[36] Ellickson PL, McCaffrey DF, Ghosh-Dastidar B, Longshore DL. New Inroads in Preventing Adolescent Drug Use: Results From a Large-Scale Trial of Project ALERT in Middle Schools. American Journal of Public Health. 2003;93(11):1830–6. PMC1448059. 14600049 10.2105/ajph.93.11.1830 PMC1448059

[37] Fisher WA, Fisher JD, Rye BJ. Understanding and promoting AIDS-preventive behavior: Insights from the theory of reasoned action. Health Psychology. 1995;14(3):255–64. 10.1037/0278-6133.14.3.255 7641667

[38] Frew PM, Archibald M, Diallo DD, Hou S-I, Horton T, Chan K, et al An Extended Model of Reasoned Action to Understand the Influence of Individual- and Network-Level Factors on African Americans’ Participation in HIV Vaccine Research. Prevention science: the official journal of the Society for Prevention Research. 2010;11(2):207–18. 10.1007/s11121-009-0162-9 PMC2858782. 20012200 PMC2858782

[39] Safren SA, Traeger L, Skeer M, O’Cleirigh C, Meade CS, Covahey C, et al Testing a social-cognitive model of HIV transmission risk behaviors in HIV-infected MSM with and without depression. Health psychology: official journal of the Division of Health Psychology, American Psychological Association. 2010;29(2):215–21. 10.1037/a0017859 PMC2841316. 20230095 PMC2841316

[40] Noar SM, Zimmerman RS. Health Behavior Theory and cumulative knowledge regarding health behaviors: are we moving in the right direction? Health Educ Res. 2005;20(3):275–90. 10.1093/her/cyg113 . 15632099

[41] Guilamo-Ramos V, Jaccard J, Dittus P, Gonzalez B, Bouris A. A Conceptual Framework for the Analysis of Risk and Problem Behaviors: The Case of Adolescent Sexual Behavior. Social Work Research. 2008;32(1):29–45. 10.1093/swr/32.1.29

[42] Link BG, Phelan J. Social Conditions As Fundamental Causes of Disease. Journal of Health and Social Behavior. 1995:80–94. 10.2307/2626958 7560851

[43] Meyer IH. Minority Stress and Mental Health in Gay Men. Journal of Health and Social Behavior. 1995;36(1):38–56. 10.2307/2137286 7738327

[44] Meyer IH. Prejudice, Social Stress, and Mental Health in Lesbian, Gay, and Bisexual Populations: Conceptual Issues and Research Evidence. Psychological bulletin. 2003;129(5):674–97. 10.1037/0033-2909.129.5.674 PMC2072932. 12956539 PMC2072932

[45] Berger I, Mooney-Somers J. Smoking Cessation Programs for Lesbian, Gay, Bisexual, Transgender, and Intersex People: A Content-Based Systematic Review. Nicotine & tobacco research: official journal of the Society for Research on Nicotine and Tobacco. 2016 Epub 2016/09/11. 10.1093/ntr/ntw216 . 27613909

[46] Lacefield K, Negy C, Schrader RM, Kuhlman C. Comparing Psychosocial Correlates of Condomless Anal Sex in HIV-Diagnosed and HIV-Nondiagnosed Men Who Have Sex with Men: A Series of Meta-Analyses of Studies from 1993–2013. LGBT health. 2015;2(3):200–20. Epub 2016/01/21. 10.1089/lgbt.2014.0069 . 26788669

[47] Mustanski B, Greene GJ, Ryan D, Whitton SW. Feasibility, Acceptability, and Initial Efficacy of an Online Sexual Health Promotion Program for LGBT Youth: The Queer Sex Ed Intervention. The Journal of Sex Research. 2015;52(2):220–30. 10.1080/00224499.2013.867924 24588408

[48] Emlet CA, Fredriksen-Goldsen KI, Kim H-J. Risk and Protective Factors Associated with Health-Related Quality of Life Among Older Gay and Bisexual Men Living With HIV Disease. The Gerontologist. 2013;53(6):963–72. 10.1093/geront/gns191 PMC3826162. 23355449 PMC3826162

[49] Ewart CK. How integrative theory building can improve health promotion and disease prevention In: Boll TJ, Frank RG, Baum A, Wallander JL, editors. Handbook of clinical health psychology: Volume 3 Models and perspectives in health psychology. 3 Washington, DC, US: American Psychological Association; 2004 p. 249–89.

[50] Ewart CK. Changing our unhealthy ways: Emerging perspectives from Social Action Theory In: DiClemente RJ, Crosby RA, Kegler MC, editors. Emerging Theories In Health Promotion Practice and Research. 2 ed. New York City, NY: Jossey-Bass; 2009 p. 359–91.

[51] Maisto SA, Ewart CK, Connors GJ, Funderburk JS, Krenek M. Use of the social competence interview and the anger transcendence challenge in individuals with alcohol use disorder. Journal of Behavioral Medicine. 2009;32(3):285–93. 10.1007/s10865-009-9201-z 19184391

[52] Bandura A. Social learning theory. Englewood Cliffs, NJ: Prentice Hall; 1997.

[53] Catania JA, Kegeles SM, Coates TJ. Towards an Understanding of Risk Behavior: An AIDS Risk Reduction Model (ARRM). Health Education Quarterly. 1990;17(1):53–72. 2318652 10.1177/109019819001700107

[54] Fishbein DH, Reuland M. Psychological correlates of frequency and type of drug use among jail inmates. Addictive Behaviors. 1994;19(6):583–98. 10.1016/0306-4603(94)90014-0 7701970

[55] Janz NK, Becker MH. The Health Belief Model: A Decade Later. Health Education Quarterly. 1984;11(1):1–47. 10.1177/109019818401100101 6392204

[56] Weintraub A, Mellins CA, Warne P, Dolezal C, Elkington K, Bucek A, et al Patterns and Correlates of Serostatus Disclosure to Sexual Partners by Perinatally-Infected Adolescents and Young Adults. AIDS and Behavior. 2017;21(1):129–40. 10.1007/s10461-016-1337-6 26874846 PMC5651172

[57] McKay MM, Alicea S, Elwyn L, McClain ZRB, Parker G, Small LA, et al The Development and Implementation of Theory-Driven Programs Capable of Addressing Poverty-Impacted Children's Health, Mental Health, and Prevention Needs: CHAMP and CHAMP+, Evidence-Informed, Family-Based Interventions to Address HIV Risk and Care. Journal of Clinical Child & Adolescent Psychology. 2014;43(3):428–41. 10.1080/15374416.2014.893519 24787707 PMC4215567

[58] Sullivan KM, Dawson Rose C, Phillips JC, Holzemer WL, Webel AR, Nicholas P, et al Sexual transmission-risk behaviour among HIV-positive persons: a multisite study using social action theory. Journal of Advanced Nursing. 2017;73(1):162–76. 10.1111/jan.13087 27485796 PMC5588908

[59] Johnson MO, Carrico AW, Chesney MA, Morin SF. Internalized heterosexism among HIV-positive, gay-identified men: implications for HIV prevention and care. Journal of consulting and clinical psychology. 2008;76(5):829 10.1037/0022-006X.76.5.829 18837600 PMC2801151

[60] Moore AR, Oppong J. Sexual risk behavior among people living with HIV/AIDS in Togo. Social science & medicine. 2007;64(5):1057–66. 17101202 10.1016/j.socscimed.2006.10.004

[61] Gore-Felton C, Rotheram-Borus M.J., Weinhardt L.S., Kelly J.A., Lightfott M., Kirshenbaum S.B., et al The healthy living project: An individually tailored, multidimensional intervention for HIV-infected persons. AIDS Education and Prevention. 2005;17(supplA):21–39. 15843115 10.1521/aeap.17.2.21.58691

[62] Lightfoot M, Rotheram-Borus MJ, Milburn NG, Swendeman D. Prevention for HIV-Seropositive Persons. Behavior Modification. 2005;29(2):227–55. 10.1177/0145445504272599 . 15657410 PMC2953374

[63] Johnson MO, Catz SL, Remien RH, Rotheram-Borus MJ, Morin SF, Charlebois E, et al Theory-Guided, Empirically Supported Avenues for Intervention on HIV Medication Nonadherence: Findings from the Healthy Living Project. AIDS Patient Care and STDs. 2003;17(12):645–56. 10.1089/108729103771928708 14746658

[64] Reynolds EK, Magidson JF, Bornovalova MA, Gwadz M, Ewart CK, Daughters SB, et al Application of the Social Action Theory to Understand Factors Associated with Risky Sexual Behavior among Individuals in Residential Substance Abuse Treatment. Psychology of addictive behaviors: journal of the Society of Psychologists in Addictive Behaviors. 2010;24(2):311–21. 10.1037/a0018929 PMC2891559. 20565157 PMC2891559

[65] Kalichman SC, Simbayi LC, Cain D, Carey KB, Carey MP, Eaton L, et al Randomized community-level HIV prevention intervention trial for men who drink in South African alcohol-serving venues. European journal of public health. 2014;24(5):833–9. Epub 2013/11/20. 10.1093/eurpub/ckt172 24248803 PMC4184338

[66] Traube DE, Holloway IW, Smith L. Theory development for HIV behavioral health: empirical validation of behavior health models specific to HIV risk. AIDS care. 2011;23(6):663–70. Epub 2011/02/25. 10.1080/09540121.2010.532532 21347886 PMC3109986

[67] Jarvis IL. Decision making under stress In: Goldberger L, Breznitz S, editors. Handbook of stress: Theoretical and clinical aspects New York City, NY: Free Press; 1982 p. 69–87.

[68] Bower GH. Mood and memory. American Psychologist. 1981;36(2):129–48. 7224324 10.1037//0003-066x.36.2.129

[69] Petty RE, Cacioppo JT. Communication and persuasion: Central and peripheral routes to attitude change. New York City, NY: Springer-Verlag; 1986.

[70] Marlatt GA, Kosturn CF, Lang AR. Provocation to anger and opportunity for retaliation as determinants of alcohol consumption in social drinkers. Journal of Abnormal Psychology. 1975;84(6):652–9. 10.1037/0021-843X.84.6.652 1194526

[71] Joormann J, Yoon KL, Zetsche U. Cognitive inhibition in depression. Applied and Preventive Psychology. 2007;12(3):128–39. 10.1016/j.appsy.2007.09.002

[72] Mauss IB, Robinson MD. Measures of emotion: A review. Cognition & emotion. 2009;23(2):209–37. 10.1080/02699930802204677 PMC2756702. 19809584 PMC2756702

[73] Traube DE, Holloway IW, Schrager SM, Kipke MD. Utilizing Social Action Theory as a Framework to Determine Correlates of Illicit Drug Use Among Young Men Who Have Sex with Men. Psychology of Addictive Behaviors. 2012;26(1):78–88. 10.1037/a0024191 PMC3241957. 21644802 PMC3241957

[74] Ford WL, Weiss G, Kipke MD, Ritt-Olson A, Iverson E, Lopez D. The Healthy Young Men’s Study: Sampling Methods to Recruit a Random Cohort of Young Men Who Have Sex with Men. Journal of gay & lesbian social services. 2009;21(4):357–73. 10.1080/10538720802498280 PMC2930784. 20823947 PMC2930784

[75] Kipke MD, Weiss G, Ramirez M, Dorey F, Ritt-Olson A, Iverson E, et al Club Drug Use in Los Angeles among Young Men Who Have Sex with Men. Substance use & misuse. 2007;42(11):1723–43. 10.1080/10826080701212261 PMC2405898. 17934992 PMC2405898

[76] MacKellar DA, Valleroy LA, Karon JM, Lemp GF, Janssen RS. The Young Men's Survey: Methods for estimating HIV seroprevalence and risk factors among young men who have sex with men. Public Health Rep. 1996;111(Supplement 1):138–44. PMC1382056 8862170

[77] Muhib F, Lin L, Steuve A, Miller R, Ford W, Johnson W, et al The Community Intervention Trial for Youth (CITY) Study team: A venue-based method for sampling hard to reach populations. Public Health Rep. 2001;116(S2):216–22. 11889287 10.1093/phr/116.S1.216 PMC1913675

[78] Radloff LS. The CES-D Scale: A Self-Report Depression Scale for Research in the General Population. Applied Psychological Measurement. 1977;1(3):385–401.

[79] Nott KH, Vedhara K. The measurement and significance of stressful life events in a cohort of homosexual HIV positive men. AIDS care. 1995;7(11):55–69. 7748911 10.1080/09540129550126966

[80] Centers for Disease Control. 2005 Youth Risk Behavior Survey. 2005.

[81] Bentler PM. On tests and indices for evaluating structural models. Personality and Individual Differences. 2007;42(5):825–9. 10.1016/j.paid.2006.09.024.

[82] Kline RB. Principles and practice of structural equation modeling. New York, NY: The Guilford Press; 2004.

[83] Muthén LK, Muthén BO. Mplus User’s Guide. Fifth Edition ed. Los Angeles, CA: Muthén & Muthén; 1998–2007.

[84] Romer D, Stanton BF. Feelings About Risk and the Epidemic Diffusion of Adolescent Sexual Behavior. Prevention Science. 2003;4(1):39–53. 10.1023/A:1021734827116 12611418

[85] Centers for Disease Control. Youth risk behavior surveillance—United States, 2009. 2010 Contract No.: SS-5.

[86] Gielen AC, McDonnell KA, Wu AW, O'Campo P, Faden R. Quality of life among women living with HIV: The importance violence, social support and self care behaviors. Social Science & Medicine. 2001;52(2):315–22. 10.1016/S0277-9536(00)00135-0 11144787

[87] Herek GM. Confronting Sexual Stigma and Prejudice: Theory and Practice. Journal of Social Issues. 2007;63(4):905–25. 10.1111/j.1540-4560.2007.00544.x

[88] Chen E, Matthews KA, Salomon K, Ewart CK. Cardiovascular reactivity during social and nonsocial stressors: Do children's personal goals and expressive skills matter? Health Psychology. 2002;21(1):16–24. 10.1037/0278-6133.21.1.16 11846340

[89] Kalichman SC. Psychological and social correlates of high-risk sexual behaviour among men and women living with HIV/AIDS. AIDS care. 1999;11(4):415–27. 10.1080/09540129947794 10533534

[90] Crepaz N, Marks G. Towards an understanding of sexual risk behavior in people living with HIV: a review of social, psychological, and medical findings. Aids. 2002;16(2):135–49. 11807297 10.1097/00002030-200201250-00002

[91] Bachanas PJ, Morris MK, Lewis-Gess JK, Sarett-Cuasay EJ, Sirl K, Ries JK, et al Predictors of Risky Sexual Behavior in African American Adolescent Girls: Implications for Prevention Interventions. Journal of Pediatric Psychology. 2002;27(6):519–30. 10.1093/jpepsy/27.6.519 12177252

[92] Donenberg GR, Emerson E, Bryant FB, Wilson H, Weber-Shifrin E. Understanding AIDS-Risk Behavior Among Adolescents in Psychiatric Care: Links to Psychopathology and Peer Relationships. Journal of the American Academy of Child & Adolescent Psychiatry. 2001;40(6):642–53. 10.1097/00004583-200106000-00008. 11392341 PMC1201503

[93] Beidas RS, Birkett M, Newcomb ME, Mustanski B. Do psychiatric disorders moderate the relationship between psychological distress and sexual risk-taking behaviors in young men who have sex with men? A longitudinal perspective. AIDS patient care and STDs. 2012;26(6):366–74. 10.1089/apc.2011.0418 22680282 PMC4932789

